# Profiling of DNA damage and repair pathways in small cell lung cancer reveals a suppressive role in the immune landscape

**DOI:** 10.1186/s12943-021-01432-5

**Published:** 2021-10-07

**Authors:** Renjing Jin, Bin Liu, Mengjun Yu, Liwei Song, Meng Gu, Ziyu Wang, Xiaobo Li, Xu Zhang, Jinghui Wang, Teng Ma

**Affiliations:** 1 Department of Cellular and Molecular Biology, Beijing Chest Hospital, Capital Medical University, Beijing Tuberculosis and Thoracic Tumor Research Institute, Beijing, 101149 China; 2 Department of Medical Oncology, Beijing Chest Hospital, Capital Medical University, Beijing Tuberculosis and Thoracic Tumor Research Institute, Beijing, 101149 China; 3 Department of Radiation Oncology, Beijing Chest Hospital, Capital Medical University/Beijing Tuberculosis and Thoracic Tumor Research Institute, Beijing, 101149 China

## Main text

Small Cell Lung Cancer accounts for nearly 15% of lung cancer incidence. Genomics alterations of *TP53* and *RB1* genes are found in almost 80% of SCLC cases. The initial treatments for SCLC are radiotherapy and/or platinum-based chemotherapy. However, a substantial number of patients diagnosed with SCLC are at risk for metastatic progression and resistance after primary treatment.

Aberrant expression of DNA damage repair gene in SCLC have been reported [1]. Mutations of DNA repair pathways are also enriched in post-treatment samples [2]. Target gene sequencing reveals that DNA damage response (DDR) pathway alterations in SCLC, both double strand breaks (DSB) and single strand breaks (SSB), have a positive correlation with high tumor mutation burden (TMB) [3]. Single-nucleotide polymorphisms (SNPs) analysis of *XRCC1* gene from the blood DNA in SCLC patients shows significant association with survival [4]. Whole-exome sequencing reveals that germline-mutated SCLC subtype favors with DNA repair-targeted therapies [5].

Activation of the immune system by blocking PD-1/PD-L1 immune checkpoint may provide the better alternative way to combat the SCLC [6–9]. Triparna Sen et al. demonstrated that PARP inhibition can activate the stimulator of interferon genes (STING) innate immune pathway in the murine SCLC model, therefore synergize with anti-PD-L1 treatment [10].

Herein, to better understand the relationship between DDR pathways and the immune landscape from patient view, we directly investigated the DDR profiling and immune landscape in SCLC patient samples. Finally, we found that the process of homologous DNA pairing and Strand Exchange in homologous recombination (HR) of doubles strand breaks (DSB) repair negatively correlates with the overall immune landscape in SCLC patients. Inhibition of RAD51-mediated DNA pairing and Strand Exchange increased the expression of checkpoint molecules expressions and migration of PBMC (peripheral blood mononuclear cell) derived from SCLC patients.

## Results and discussion

**Fig. 1:**
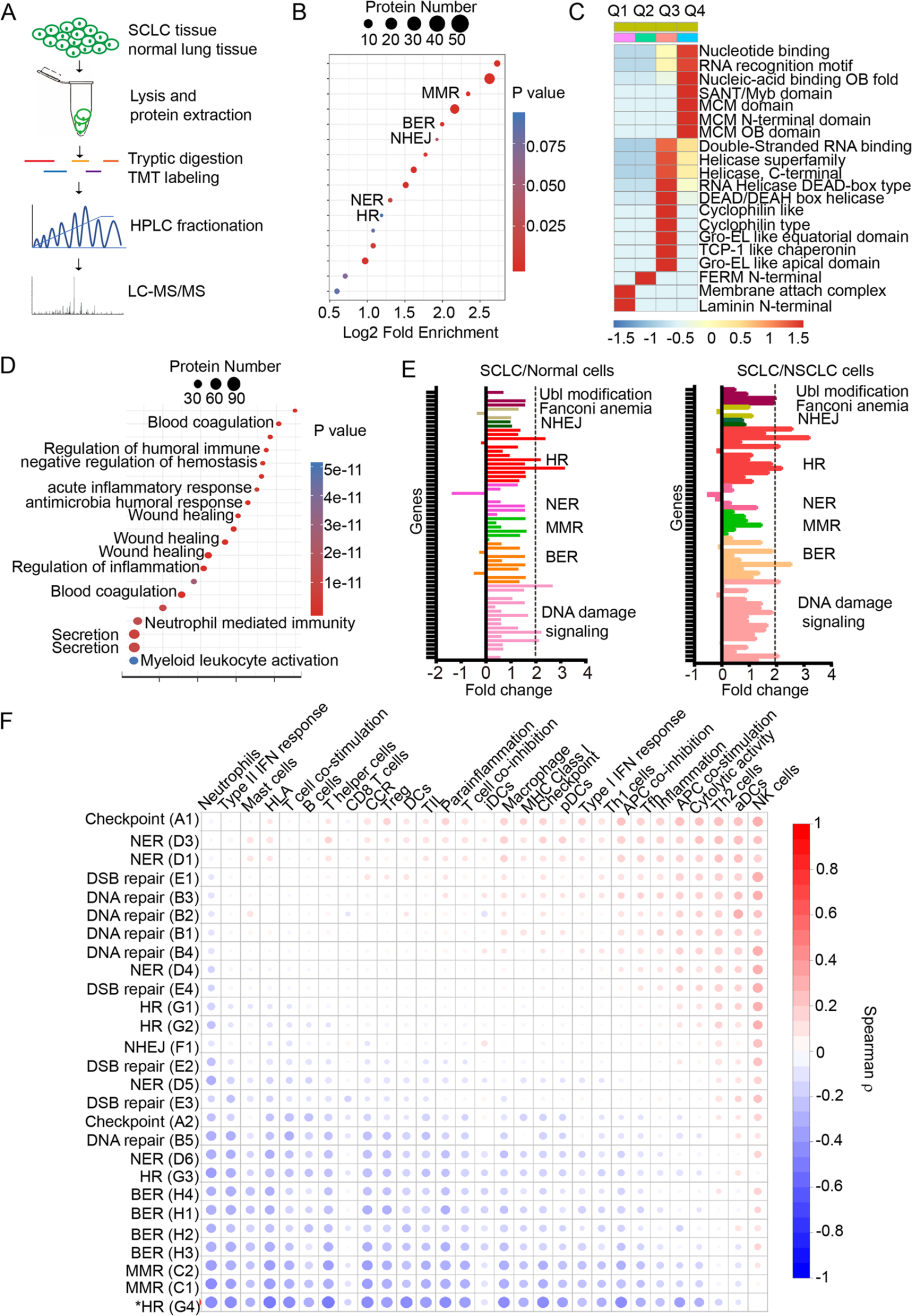
DDR profiling and correlation with immune landscape in SCLC. **A**, flowchart of mass spectrometry analysis of SCLC tissues and normal controls. **B**, different DNA repair pathways were enriched in the upregulated proteome of SCLC. **C**, the classification of domains from upregulated proteins in SCLC. **D**, the KEGG analysis of downregulated pathways in SCLC. **E**, qPCR array analysis of differentially expressed DNA damage and repair genes in SCLC cell versus BEP2D cells and NSCLC cells. **F**, Spearman ρ correlation matrix between 27 DDR pathways and immune pathways and immune cells

To gain an insight of the protein expression profiles in SCLC patients, comparative proteomic analysis of SCLC tissues and the counterpart normal lung tissues was performed using LC-MS/MS as depicted in Fig. 1A. To dissect the functional enrichment of upregulated and downregulated proteins respectively, Encyclopedia of Genes and Genomes (KEGG) database was used to identify enriched pathways by a two-tailed Fisher’s exact test. As shown in Fig. 1B, 5 canonical DNA repair pathways including MMR (Mismatch Repair), BER (Base Excision Repair), NHEJ (Non Homologous End Joining), HR (Homologous Recombination) and NER (Nucleotide Excision Repair) were enriched among upregulated proteins. Similarly, protein domain and molecular function enrichment analysis showed that the DNA binding domains were enriched in the Q4 group which were upregulated with fold change > 2 (Fig. 1C). Meanwhile, the protein function enrichment for the downregulated proteins in SCLC tissues were also analyzed (Fig. 1D). The functions of the downregulated proteins were exclusively enriched extracellularly including the inflammation and immune related pathways, indicating a suppressive tumor immune microenvironment in SCLC.

Next, to further validate the observations of DDR pathway change in SCLC/normal tissues, DDR gene expressions were further compared using 83 DDR genes qPCR array among BEP2D (human normal bronchial epithelia cells), A549 and H1299 (NSCLC cell lines), H446 and H69 (SCLC cell lines). The expression pattern demonstrated that a number of DDR genes had an increased expression in SCLC cells (Fig. 1E).

To investigate the correlations between DDR pathways and immune landscape in SCLC, we first investigated the patient-level DDR pathway profiling using GSEA algorithm based on published RNA-Seq data [11]. Gene expression data was converted into DDR pathway expression data individually for each patient (Supplementary Fig. 1A and B). Canonical DDR pathway gene sets from the curated KEGG and Reactome collections were used (Supplementary Table 1). The correlations of different pathways were also analyzed (Supplementary Fig. 1C).

For the immune landscape analysis in SCLC, hallmark pathway gene lists were downloaded from the mSigDB website for pathway level analysis. The immune content score was calculated using immune specific genes from literature [12]. CIBERSORT was used to estimate the relative proportion of different immune cell types [13].

To dissect which DDR pathways correlated with the immune landscape of SCLC, we performed correlation analysis between different DDR pathways and the different immune contents in SCLC patients. The results showed that homologous DNA pairing and strand exchange pathway (labeled as G4) negatively correlated with the immune content (Fig. 1F).

**Fig. 2:**
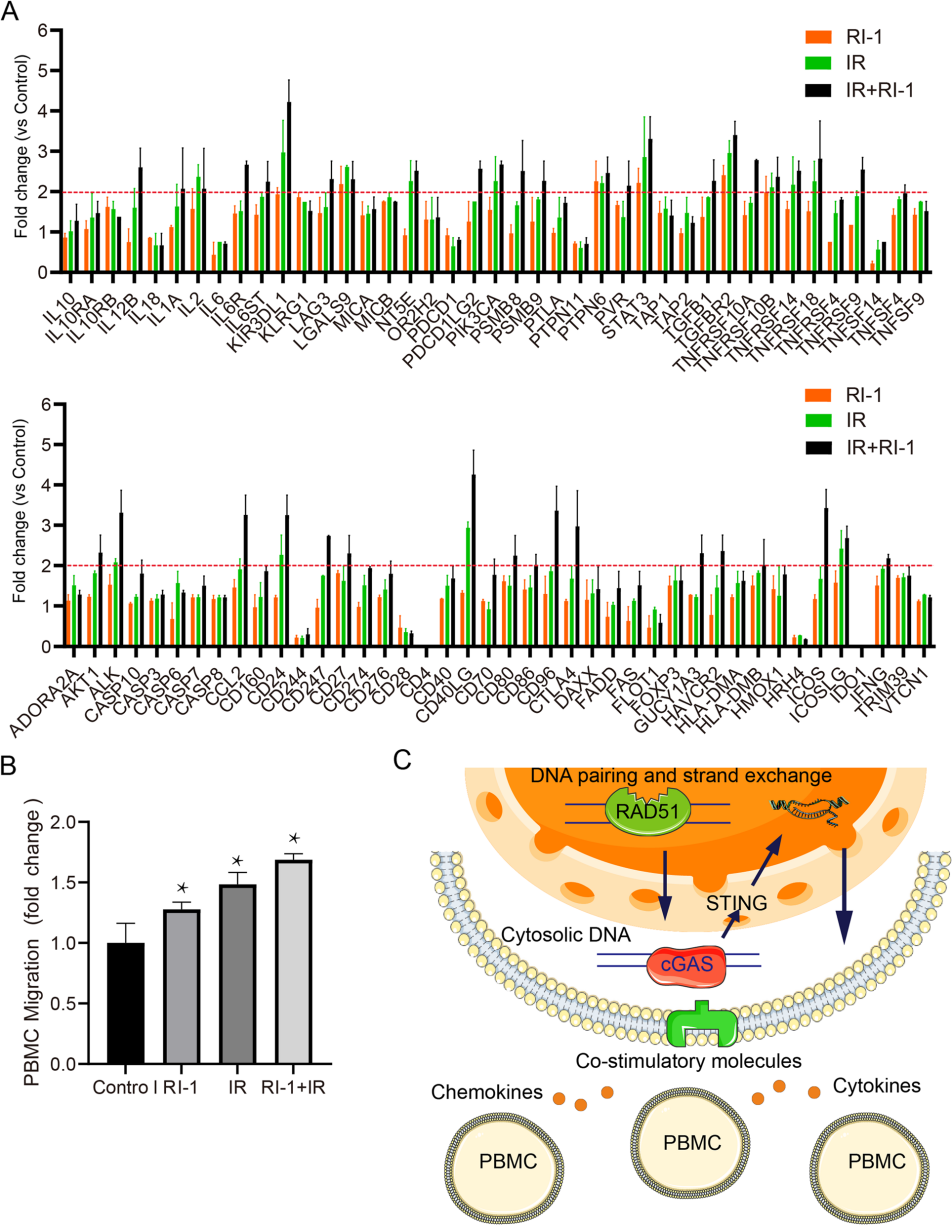
Targeting DNA pairing and strand exchange process activated the immune contents of SCLC. **A**, RAD51 inhibition primed SCLC cells to immune checkpoint activation. H446 cells were pretreated with 10 μM RAD51 inhibitor RI-1 for 6 h, then followed by 4Gy IR treatment, 12 h later cells were harvested and RNAs were extracted for qPCR array. Eighty-three genes related to immune checkpoint were amplified by qPCR. **B.** RAD51 inhibition promoted the migration PBMCs derived from SCLC patients. Migration assay was performed using Transwell chambers. PBMC from SCLC patients were seeded into the upper wells, while the lower chambers contained either RPMI medium or conditioned medium from H446 cells treated with RI-1 or in combination with IR. The results of these experiments are expressed as fold-change vs. control± SEM after 6 h incubation (*n* = 4). Statistical analysis was performed by ANOVA (*P < 0.05). **C**. A schematic model of how targeting RAD51 primes the activation of immune contents in the tumor microenvironment. RAD51 mediated DNA pairing and strand exchange interconnects between DSB repair and immune responses

Since RAD51 is the critical player in DNA pairing and strand exchange process of HR, we further investigated if RAD51 inhibition could affect the immune checkpoint molecules expressions after DNA damage. A 6 MV X-ray photon beam was used to generate DNA lesions in both H446 cells and H526 cells. The cells were treated with 10 μM RAD51 specific inhibitor RI-1 which abrogated the RAD51 foci but not its binding partners BRCA1 and BRCA2 foci followed by 4Gy ionizing radiation (Supplementary Fig. 2), and 83 mRNAs relative with immune checkpoint were amplified (Fig. 2A and Supplementary Fig. 3). The results indicated that several important molecules such as *IL12B*, *CD40LG*, *ICOS* were upregulated, demonstrating an important role of RAD51 in suppression of immune content in SCLC.

To further investigate the role of RAD51-mediated DNA pairing and strand exchange in suppression of immune cells, we performed assays to evaluate whether the conditioned medium of RAD51 inhibitor Rl-I treated H446 and H526 cells had a chemotactic effect on PBMCs from SCLC patients. We observed that the number of migrated cells increased in conditioned medium from Rl-1 treated H446 and H526 cells compared with the RPMI1640 medium alone. When combined with 4Gy IR, the conditioned medium significantly increased PBMC migration (Fig. 2B and Supplementary Fig. 3C) (*P* < 0.05). The increased PBMC migration was not due to radiation-induced cell death (Supplementary Fig. 4). Mechanistically, cytosolic DNA sensing pathway was shown to be activated after RAD51 inhibition, as manifested by increased dsDNA-cGAS staining (Supplementary Fig. 5).

Furthermore, in multiple-plex immunofluorescence staining of RAD51/CD4/CD8/CD20/Foxp3 in SCLC tissue microarray, SCLC tissues with increased RAD51 staining showed decreased CD8+ lymphocyte infiltration (Supplementary Fig. 6). Collectively, these observations indicate that the DNA pairing and strand exchange process in HR confers suppressive signal to induce immune cell migration in SCLC patients.

The intrinsic or extrinsic insults on genome will cause micronuclei formation and release of cytosolic DNA, the latter of which stimulates the cGAS-STING pathway and induce the expression of type I interferon [14]. Here we demonstrated the suppressive role of RAD51-mediated DNA pairing and strand exchange process in SCLC immune microenvironment. However, other genome instability events such as Microsatellite instability (MSI) and other DDR pathways such as MMR may also contribute to the suppressive SCLC immune microenvironment. The SCLC cells defective in DNA pairing and strand exchange after radiation may have altered secretome and exosome pattern, which subsequently affect the immune cells infiltration.

The dysregulated DDR gene expressions could mainly result from a double loss of *RB1* and *TP53* gene in SCLC which are the hallmark genomic aberrations. The loss of Rb activity in SCLC will lead to increased expressions of E2F1-target genes in SCLC, such as DNA repair genes and PARP1 [15], an E2F1 co-activator which has been investigated as a therapeutic target in SCLC partially through upregulation of PD-L1 expression [1, 7, 9].

In summary, here we report RAD51 plays important functions in SCLC immune microenvironment, further demonstrating the combination of DDR inhibition and Immune checkpoint as promising therapeutic potential in treating SCLC.

## Supplementary Information

**Additional file 1: Supplementary Table 1.** DNA damage and repair pathways. **Figure S1.** Patient-level profiling of DDR pathways. (A) RNA-Seq data is converted into (B) pathway enrichment data. Expression is median-scaled and ranked across all samples gene by gene. (B) Gene Set Enrichment Analysis pre-ranked on gene ranks generates pathway enrichment NES, and hierarchical clustering generates a pathway heatmap. (C) Spearman ρ correlation matrix of intercorrelation among the pathways across all samples. DDR, DNA damage and Repair; DSB, double-strand break; BER, base excision repair; NER, nucleotide excision repair; HR, homologous recombination repair; NHEJ, non-homologous end joining; GSEA, Gene Set Enrichment Analysis; The blue box indicated the correlation matrix of MMR and other pathways. **Figure S2.** RAD51 inhibitor specifically abrogated RAD51 foci. (A) H446 or H526 cells were pretreated with 10 μM RI-1 and followed by 4Gy IR. Six hours after treatment, the RAD51 foci was stained and summarized. (B) and (C) H446 or H526 cells were treated the same way as above. The BRCA1 foci and BRCA2 foci were stained and summarized. The results are represented as Mean ± SEM (*n* = 3). * *P* < 0.05, ** *P* < 0.01, *** *P* < 0.001. **Figure S3.** Impact of RAD51 inhibition in H526 cells. (A) The relative DDR genes expression profile in H526 cells. (B) RAD51 inhibition increased immune checkpoint molecules expressions in H526 cells. H526 cells were pretreated with 10 μM RAD51 inhibitor RI-I for 6 h, then followed by 4Gy IR, 12 h later cells were harvested and RNAs were extracted for qPCR array. Eighty-three genes related to immune checkpoint were amplified by qPCR. (C) RAD51 inhibition promoted the migration of PBMCs derived from SCLC patients. Migration assay was performed using Transwell chambers. PBMC from SCLC patients were seeded into the upper wells, while the lower chambers contained either RPMI medium or conditioned medium from H526 cells treated with RI-1 or in combination with IR for 12 h. The results of these experiments are expressed as fold-change vs. control± SEM after 6 h incubation (*n* = 4). Statistical analysis was performed by ANOVA (*P < 0.05). **Figure S4.** RAD51 mediated PBMC migration effect was not due to cell death. (A) H446 or H526 cells were pretreated with 10 μM RI-1 and followed by 4Gy IR. The cell viability assay was performed at 0 h, 12 h and 24 h after treatment. The cell viability percentage relative to control was summarized. (B) H446 or H526 cells were pretreated with 10 μM RI-1 and followed by 4Gy IR. Quantitative analysis of live, early, and late apoptosis and cell death at 0 h, 12 h and 24 h was performed with Muse^@^ Annexin V & Dead Cell Kit. (C) H446 or H526 cells were pretreated with 10 μM RI-1 and 10 μM Z-VAD-FMK or 1 μM MRT68921 and followed by 4Gy IR. The PBMC migration was evaluated. **Figure S5.** RAD51 inhibition activated the dsDNA-cGAS-STING pathway in SCLC cells. (A) H446 or H526 cells were pretreated with 10 μM RI-1 and followed by 4Gy IR. Six hours after treatment, the STING expression was stained and summarized. (B) H446 or H526 cells were pretreated with 10 μM RI-1 and followed by 4Gy IR. Six hours after treatment, the dsDNA and cGAS were co-stained and summarized. * *P* < 0.05, ** *P* < 0.01, *** *P* < 0.001. **Figure S6.** Multiplex immunofluorescence staining of RAD51, CD4, CD8, FOXP3 and CD20. (A) The overview of the stained SCLC tissue microarray. Normal lung tissues and SCLC tissues were labeled. (B) The representative HE staining and immunofluorescence staining images of SCLC tissues with high or low infiltrated lymphocytes. (C) Summary of cytoplasmic and nucleus staining of RAD51. The right panel showed the correlation between cytoplasm and nucleus expression of RAD51. (D) Summary of CD8+ cell density in SCLC tissues with RAD51 high and low expression.

## Data Availability

All data are available in the main text or the supplementary materials.

## Abbreviations

SCLC: Small Cell Lung Cancer
DDR: DNA Damage Response
DSB: Double Strand Break
SSB: Single Strand Break
TMB: Tumor Mutation Burden
SNPs: Single-nucleotide polymorphisms
PARP: Poly (ADP-ribose) polymerase
STING: Stimulator of interferon genes
cGAS: Cyclic GMP–AMP synthase
HR: Homologous Recombination
NHEJ: Non-homologous End Joining
BER: Base excision repair
MMR: Mismatch Repair
NER: Nucleotide Excision Repair
PBMC: Peripheral Blood Mononuclear Cell
