# Supplementary material for: Profiling of DNA damage and repair pathways in small cell lung cancer reveals a suppressive role in the immune landscape
Source: Mol Cancer. 2021 Oct 7;20:130. doi: 10.1186/s12943-021-01432-5 (PMC8496044; doi:10.1186/s12943-021-01432-5)
Supplement: Supplementary file 1 — Additional file 1: Supplementary Table 1. DNA damage and repair pathways. Figure S1. Patient-level profiling of DDR pathways. (A) RNA-Seq data is converted into (B) pathway enrichment data. Expression is median-scaled and ranked across all samples gene by gene. (B) Gene Set Enrichment Analysis pre-ranked on gene ranks generates pathway enrichment NES, and hierarchical clustering generates a pathway heatmap. (C) Spearman ρ correlation matrix of intercorrelation among the pathways across all samples. DDR, DNA damage and Repair; DSB, double-strand break; BER, base excision repair; NER, nucleotide excision repair; HR, homologous recombination repair; NHEJ, non-homologous end joining; GSEA, Gene Set Enrichment Analysis; The blue box indicated the correlation matrix of MMR and other pathways. Figure S2. RAD51 inhibitor specifically abrogated RAD51 foci. (A) H446 or H526 cells were pretreated with 10 μM RI-1 and followed by 4Gy IR. Six hours after treatment, the RAD51 foci was stained and summarized. (B) and (C) H446 or H526 cells were treated the same way as above. The BRCA1 foci and BRCA2 foci were stained and summarized. The results are represented as Mean ± SEM (n = 3). * P < 0.05, ** P < 0.01, *** P < 0.001. Figure S3. Impact of RAD51 inhibition in H526 cells. (A) The relative DDR genes expression profile in H526 cells. (B) RAD51 inhibition increased immune checkpoint molecules expressions in H526 cells. H526 cells were pretreated with 10 μM RAD51 inhibitor RI-I for 6 h, then followed by 4Gy IR, 12 h later cells were harvested and RNAs were extracted for qPCR array. Eighty-three genes related to immune checkpoint were amplified by qPCR. (C) RAD51 inhibition promoted the migration of PBMCs derived from SCLC patients. Migration assay was performed using Transwell chambers. PBMC from SCLC patients were seeded into the upper wells, while the lower chambers contained either RPMI medium or conditioned medium from H526 cells treated with RI-1 or in combination with IR for 12 [file 12943_2021_1432_MOESM1_ESM.docx]

**Profiling of DNA damage and repair pathways in Small Cell Lung Cancer reveals a suppressive role in the immune landscape**

**Supplementary Information**

**Materials and Methods………………………………………………………………7**

**Table S1……………………………………………………………………………….8**

**Figure S1……………………………………………………………………………...9**

**Figure S2…………………………………………………………………………….10**

**Figure S3…………………………………………………………………………….12**

**Figure S4…………………………………………………………………………….13**

**Figure S5…………………………………………………………………………….14**

**Figure S6…………………………………………………………………………….15**

**Material and Methods**

**Patient Samples**

Pathologically verified SCLC and paired normal lung tissues were purchased from the Biobank of Shanghai OUTDO Biotech CO., LTD. Processing of the samples were approved by the ethics committee of Biobank of Shanghai OUTDO Biotech CO., LTD. The assigned study/project number is YB M-05-01.

**Protein extraction and trypsin digestion**

The tissue samples are taken from -80 °C and ground thoroughly to powder with liquid nitrogen. Then 4X volumes of lysis buffer was added to the tissue powder (8M urea, 1% protease inhibitor, 3μM TSA and 50mM NAM). Samples were sonicated. The remaining debris was removed by centrifuge at 12,000g at 4℃ for 10min. The supernatant was collected and the protein concentration was determined with a BCA kit according to the manufacturer’s instructions. For trypsin digestion, the protein supernatant was treated with 5mM dithiothreitol (DTT) for 30min at 56℃ and alkylated with 11mM iodoacetamide for 15min at room temperature in darkness. The protein samples were then diluted with 100mM triethylammonium bicarbonate (TEAB) to urea concentration of less than 2M. Finally, trypsin was added at 1:50 mass ration of trypsin/protein and proteins were digested overnight. A second 4h digestion was performed with trypsin/protein at 1:100 mass ration.

**TMT labeling**

After trypsin digestion, peptide was desalted by Strata X C18 SPE column (Phenomenex) and vacuum-dried. Peptide was reconstituted in 0.5 M TEAB and processed according to the manufacturer’s protocol for TMT kit. Briefly, one unit of TMT reagent were thawed and reconstituted in acetonitrile. The peptide mixtures were then incubated for 2 h at room temperature and pooled, desalted and dried by vacuum centrifugation.

**LC-MS/MS analysis**

The tryptic peptides were dissolved in 0.1% formic acid (solvent A), directly loaded onto a home-made reversed-phase analytical column (15-cm length, 75 μm i.d.). The gradient was comprised of an increase from 6% to 23% solvent B (0.1% formic acid in 98% acetonitrile) over 26 min, 23% to 35% in 8 min and climbing to 80% in 3 min then holding at 80% for the last 3 min, all at a constant flow rate of 400 nL/min on an EASY-nLC 1000 UPLC system.

The peptides were subjected to NSI source followed by tandem mass spectrometry (MS/MS) in Q Exactive^TM^ Plus (Thermo) coupled online to the UPLC. The electrospray voltage applied was 2.0 kV. The m/z scan range was 350 to 1800 for full scan, and intact peptides were detected in the Orbitrap at a resolution of 70,000. Peptides were then selected for MS/MS using NCE setting as 28 and the fragments were detected in the Orbitrap at a resolution of 17,500. A data-dependent procedure that alternated between one MS scan followed by 20 MS/MS scans with 15.0s dynamic exclusion. Automatic gain control (AGC) was set at 5E4. Fixed first mass was set as 100 m/z.

**Database search**

The resulting MS/MS data were processed using Maxquant search engine (v.1.5.2.8). Tandem mass spectra were searched against human uniprot database concatenated with reverse decoy database. Trypsin/P was specified as cleavage enzyme allowing up to 4 missing cleavages. The mass tolerance for precursor ions was set as 20 ppm in First search and 5 ppm in Main search, and the mass tolerance for fragment ions was set as 0.02 Da. Carbamidomethyl on Cys was specified as fixed modification and Acetylation modification and oxidation on Met were specified as variable modifications. FDR was adjusted to < 1% and minimum score for modified peptides was set > 40.

**Bioinformatics analysis**

Gene Ontology (GO) annotation proteome was derived from the UniProt-GOA database ( <http://www.ebi.ac.uk/GOA/>). Identified proteins domain functional description were annotated by InterProScan (a sequence analysis application) based on protein sequence alignment method, and the InterPro (<http://www.ebi.ac.uk/interpro/>) domain database was used. Kyoto Encyclopedia of Genes and Genomes (KEGG) database was used to annotate protein pathway. We used wolfpsort, a subcellular localization predication soft to predict subcellular localization. Soft MoMo (motif-x algorithm) was used to analysis the model of sequences constituted with amino acids in specific positions of modify-21-mers (10 amino acids upstream and downstream of the site). Enrichment of GO or KEGG analysis was done by a two-tailed Fisher’s exact test to test the enrichment of the differentially modified proteins.

**Cell lines and treatment**

Human bronchial epithelial cell line (BEP2D) cells were obtained from Dr CC Harris (Laboratory of Human Carcinogenesis Division of Basic Science, National Cancer Institute, NIH, USA). The cells were maintained in serum-free LHC-8 medium (Gibco, USA) supplemented with 100 units per ml of penicillin and 100 μg/ml of gentamycin in a humidified incubator at 37 °C with 5% CO_2_. Two NSCLC cell lines (A549 and H1299) and three SCLC cell lines (H446, H69 and H526) were grown in RPMI supplemented with 10% FBS under 37 °C with 5% CO_2_ condition. RAD51 inhibitor Rl-1(Cat# S8077), Z-VAD-FMK (Cat# S7023) and MRT68921 HCL (Cat# S7949) were purchased from Selleck. To generate DNA damage in cells, a 6 MV X-ray photon beam was used to treat H446 or H526 cells at indicated doses.

**Quantitative Reak-Time PCR (RT-qPCR)**

RNAs from the indicated cell lines were extracted according to standard procedure. Then cDNAs were synthesized with 5×PrimeScript RT Master Mix（Perfect Real Time）(Cat. # RR036A, Takara Bio Inc. Japan). Then RT-qPCR was applied to detect either the DNA damage repair genes or immune checkpoint genes at Applied Biosystems 7500 using qPCR arrays (WCGENE, Shanghai, China).

**Patient-level DDR pathways profiling and the immune landscape analysis**

Patient-level DDR pathways profiling and the immune landscape analysis were conducted based on published RNA-Seq data including 77 SCLC patients^11^. For DDR pathway profiling, individual patient gene rank files were used as input for the Gene Set Enrichment Analysis (GSEA) pre-ranked algorithm with 1000 permutations to generate individual patient DDR gene set profiles with normalized enrichment scores. The GSEA gene sets used are standard curated pathways from KEGG and Reactome in GSEA mSigDB website (Supplementary Table 1).

For the immune landscape analysis, hallmark pathway gene lists were downloaded from the mSigDB website for pathway level analysis. The immune content score was calculated using immune specific genes from literature. Cibersort was used to estimate the relative proportion of different immune cell types.

**Immunofluorescence staining**

Cells cultured on glass coverslips were treated as indicated in the figure legends. After washing with PBS, cells were fixed in 4% paraformaldehyde for 15 minutes, and permeabilized in 0.3% triton X-100 for 10 minutes at room temperature. Then the cells were blocked with 10% FBS in PBS and incubated with primary antibodies for 2 hours. Subsequently, samples were washed 3 times with PBS and incubated with corresponding secondary antibodies for 1 hour. The following antibodies were used: cGAS (D1D3G) Rabbit mAb (Cell Signaling; Cat # 15102); STING (D2P2F) Rabbit mAb (Cell Signaling; Cat # 13647); BRCA1 Antibody (D-9) (Santa Cruz; Cat #: sc-6954); BRCA2 antibody (Abcam; Cat # ab27976); dsDNA antibody (Abcam; Cat #27156); RAD51 (Abcam; Cat # ab133534); ProLong Gold Antifade Mountant (Thermo Fisher Scientific; P36930) was used to stain nuclear DNA. Coverslips were mounted onto glass slides and visualized using a Nikon ECLIPSE E800 fluorescence microscope.

**SCLC Tissue Microarrays**

Human SCLC tissue microarrays (TMA) were purchased from Alenabio (DC-Lun01093). All detailed clinical information including pathology, diagnosis and stage are freely available on the Web (<http://www.avilabio.com/public/details?productId=59670&searchText=>).

**Immunohistochemistry staining**

After baking and dewaxing, the SCLC tissue microarrays were subjected to antigen retrieval in boiled pH9.0 Tris-EDTA buffer for 15min. After cooling and washing with PBS, 3% H_2_O_2_ was used to block the endogenous (horseradish peroxidase) HRP for 15min. Then the TMAs were sequentially incubated with goat serum for 30min, with indicated primary antibodies at 4℃ overnight and with secondary antibodies at 37℃ for 30min. For multiplex staining, TSA-Fluorescein was added at 37℃ for 10min. The following antibodies were used: CD4 (Maxim, RMA-0620), CD8 (Maxim, RMA-0514), CD20 (Abcam; Cat # ab9475), FOXP3 (Cell Signaling; Cat # 98377), RAD51 (Abcam; Cat # ab133534). The fluorescents for CD4, CD20, FOXP3, CD8 and RAD51 are Opal 520, Opal 540, Opal 570, Opal 650 and Opal 690 respectively. After staining nucleus with DAPI, the images and results were captured and analyzed by PE Vectra automated multispectral histopathology quantitative analysis system (Perkin Elmer)

**Cell Viability Assay**

The Cell Counting Kit-8 (CCK-8; Dojindo Molecular Technologies, Kumamoto, Japan) assay was performed to investigate the viability of SCLC cells treated as indicated according to the manufacturer’s instructions. After different treatment, H446 and H526 cells were seeded in 96-well plates with 4000 cells per well. Then, 10 μL of CCK-8 reagent was added to each well of 96-well plates at 0h, 12h and 24 h respectively. The plates were incubated for 2 hours at 37°C. The absorbance at 450 nm of each well were measured with an EL-800 Universal Microplate Reader (BioTek Instruments, Inc., Winooski, VT, USA).

**Cell Apoptosis Detection**

For cell apoptosis detection after RI-1 or IR treatment or combinational treatment, the Muse® Annexin V & Dead Cell Kit (Luminex, Part number: MCH100105, Austin, U.S.A) was used according to the instructions. Briefly, the 100μL treated cells suspension was prepared in 1.5ml Eppendorf tube and mixed with 100 μL of Muse® Annexin V & Dead Cell Reagent. The cells were incubated for 20 minutes at room temperature and then subjected to Guava^@^ Muse^@^ Cell Analyzer (Luminex, Austin, U.S.A).

**PBMC migration assay**

The SCLC-derived PBMCs were prepared by Ficoll density gradient centrifugation in accordance with the hospital ethics committee. The PBMC migration assay was performed in the 24-well transwell chamber (Corning Inc., Tewksbury, USA) and cell culture inserts containing filters with a pore-size of 5 μm (Corning Inc., Tewksbury, USA) following the manufacturer's instructions. The bottom chambers contained the conditioned medium from SCLC cells after different treatments as indicated. Transwells were incubated at 37 °C in 5% CO2 for 6 h. The number of cells migrating into the bottom chamber was determined by hemocytometer.

**Statistical analysis**

The results are expressed as mean±standard error of mean and were calculated from quantita­tive data obtained from three replicate experiments. Statistical analysis was performed using one‑way analysis of variance (ANOVA) in SPSS v17.0 software. The significance of the differences between two groups were used Student’s t-test. The p-values ≤0.05 were considered significant.

**Supplementary Table 1. DNA damage and repair pathways.**

| **Gene Set** | **Pos.** | **Pathway** |
| --- | --- | --- |
| REACTOME_G1_S_DNA_DAMAGE_CHECKPOINTS | A1 | Checkpoint |
| REACTOME_G2_M_DNA_DAMAGE_CHECKPOINT | A2 | Checkpoint |
| REACTOME_DNA_REPAIR | B1 | Repair |
| REACTOME_TP53_REGULATES_TRANSCRIPTION_OF_DNA_REPAIR_GENES | B2 | Repair |
| REACTOME_DNA_DAMAGE_BYPASS | B3 | Repair |
| REACTOME_RECOGNITION_OF_DNA_DAMAGE_BY_PCNA_CONTAINING_REPLICATION_COMPLEX | B4 | Repair |
| REACTOME_SUMOYLATION_OF_DNA_DAMAGE_RESPONSE_AND_REPAIR_PROTEINS | B5 | Repair |
| REACTOME_MISMATCH_REPAIR | C1 | MMR |
| KEGG_MISMATCH_REPAIR | C2 | MMR |
| REACTOME_NUCLEOTIDE_EXCISION_REPAIR | D1 | NER |
| REACTOME_DNA_DAMAGE_RECOGNITION_IN_GG_NER | D2 | NER |
| REACTOME_GLOBAL_GENOME_NUCLEOTIDE_EXCISION_REPAIR_GG_NER | D3 | NER |
| REACTOME_GAP_FILLING_DNA_REPAIR_SYNTHESIS_AND_LIGATION_IN_GG_NER | D4 | NER |
| REACTOME_TRANSCRIPTION_COUPLED_NUCLEOTIDE_EXCISION_REPAIR_TC_NER | D5 | NER |
| KEGG_NUCLEOTIDE_EXCISION_REPAIR | D6 | NER |
| REACTOME_DNA_DOUBLE_STRAND_BREAK_RESPONSE | E1 | DSB |
| REACTOME_DNA_DOUBLE_STRAND_BREAK_REPAIR | E2 | DSB |
| REACTOME_DNA_DAMAGE_TELOMERE_STRESS_INDUCED_SENESCENCE | E3 | DSB |
| REACTOME_PROCESSING_OF_DNA_DOUBLE_STRAND_BREAK_ENDS | E4 | DSB |
| REACTOME_NONHOMOLOGOUS_END_JOINING_NHEJ | F1 | NHEJ |
| REACTOME_HDR_THROUGH_HOMOLOGOUS_RECOMBINATION_HRR | G1 | HR |
| REACTOME_HOMOLOGY_DIRECTED_REPAIR | G2 | HR |
| KEGG_HOMOLOGOUS_RECOMBINATION | G3 | HR |
| REACTOME_HOMOLOGOUS_DNA_PAIRING_AND_STRAND_EXCHANGE | G4 | HR |
| REACTOME_BASE_EXCISION_REPAIR | H1 | BER |
| REACTOME_BASE_EXCISION_REPAIR_AP_SITE_FORMATION | H2 | BER |
| REACTOME_PCNA_DEPENDENT_LONG_PATCH_BASE_EXCISION_REPAIR | H3 | BER |
| KEGG_BASE_EXCISION_REPAIR | H4 | BER |


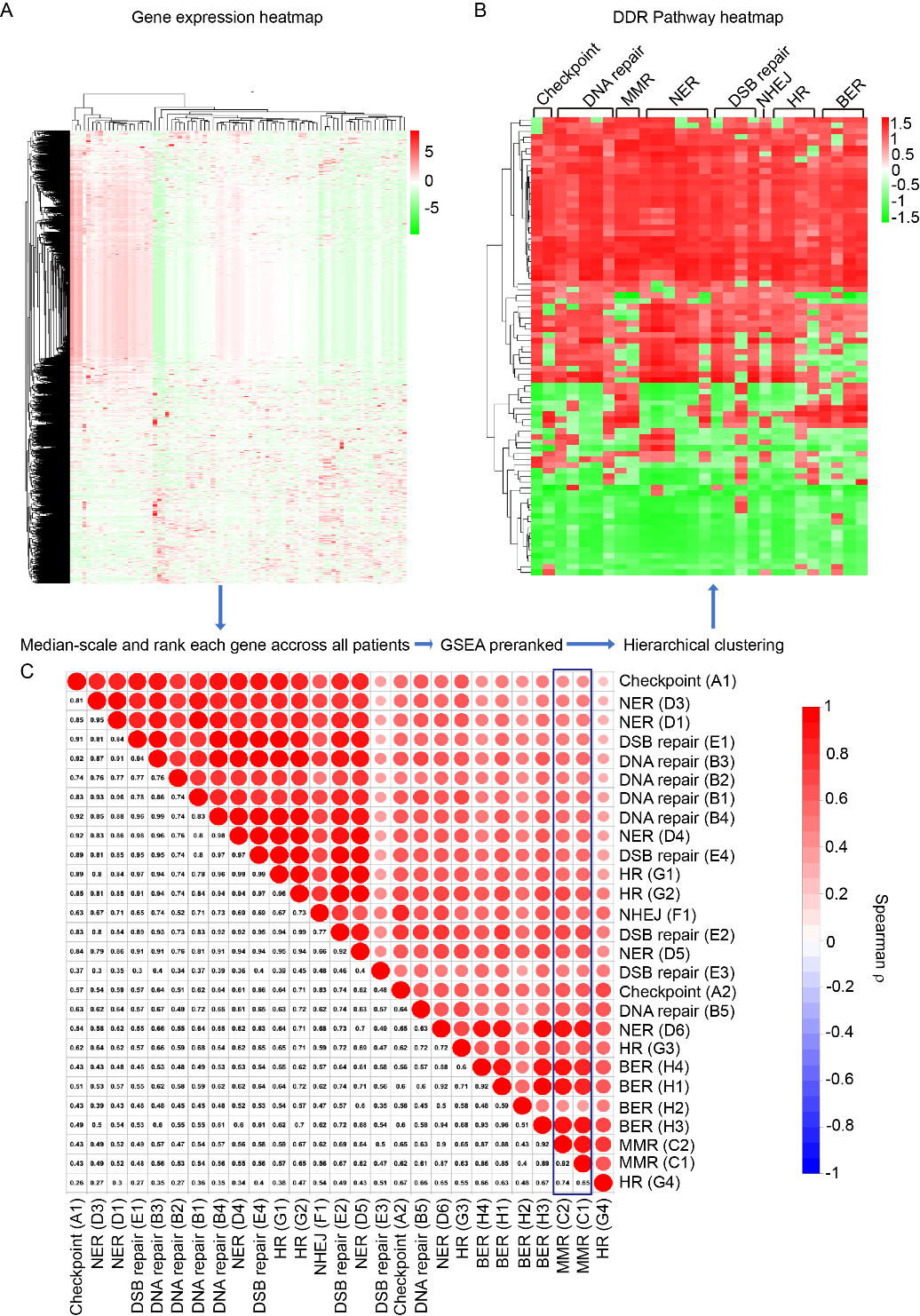


Figure S1. Patient-level profiling of DDR pathways.

**(A)** RNA-Seq data is converted into (B) pathway enrichment data. Expression is median-scaled and ranked across all samples gene by gene. **(B)** Gene Set Enrichment Analysis pre-ranked on gene ranks generates pathway enrichment NES, and hierarchical clustering generates a pathway heatmap.

**(C)** Spearman ρ correlation matrix of intercorrelation among the pathways across all samples. DDR, DNA damage and Repair; DSB, double-strand break; BER, base excision repair; NER, nucleotide excision repair; HR, homologous recombination repair; NHEJ, non-homologous end joining; GSEA, Gene Set Enrichment Analysis; The blue box indicated the correlation matrix of MMR and other pathways.


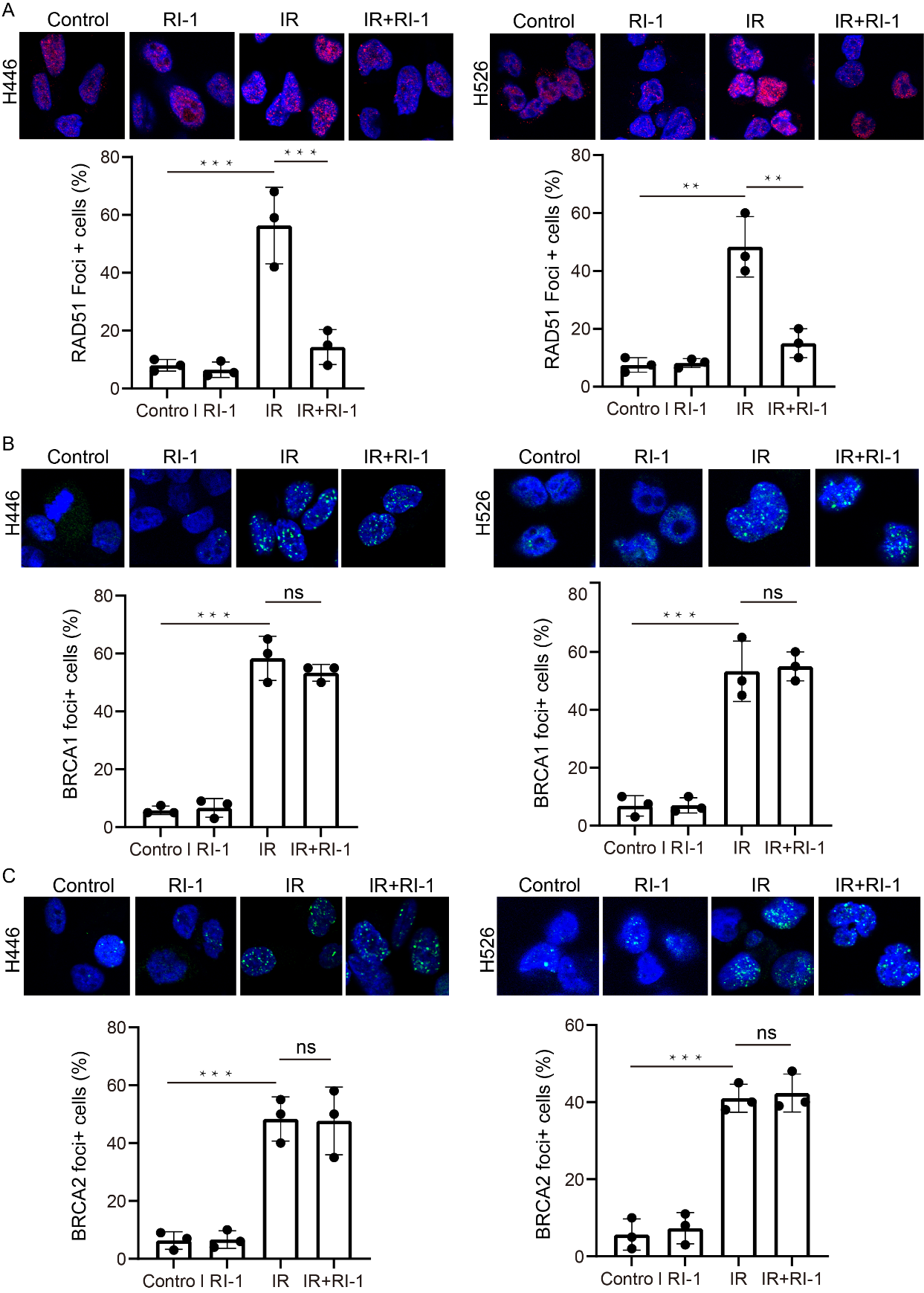


Figure S2. RAD51 inhibitor specifically abrogated RAD51 foci. **(A)** H446 or H526 cells were pretreated with 10μM RI-1 and followed by 4Gy IR. Six hours after treatment, the RAD51 foci was stained and summarized. **(B)** and **(C)** H446 or H526 cells were treated the same way as above. The BRCA1 foci and BRCA2 foci were stained and summarized. The results are represented as Mean ± SEM (n=3). * *P* <0.05, ** *P* <0.01, *** *P* <0.001.


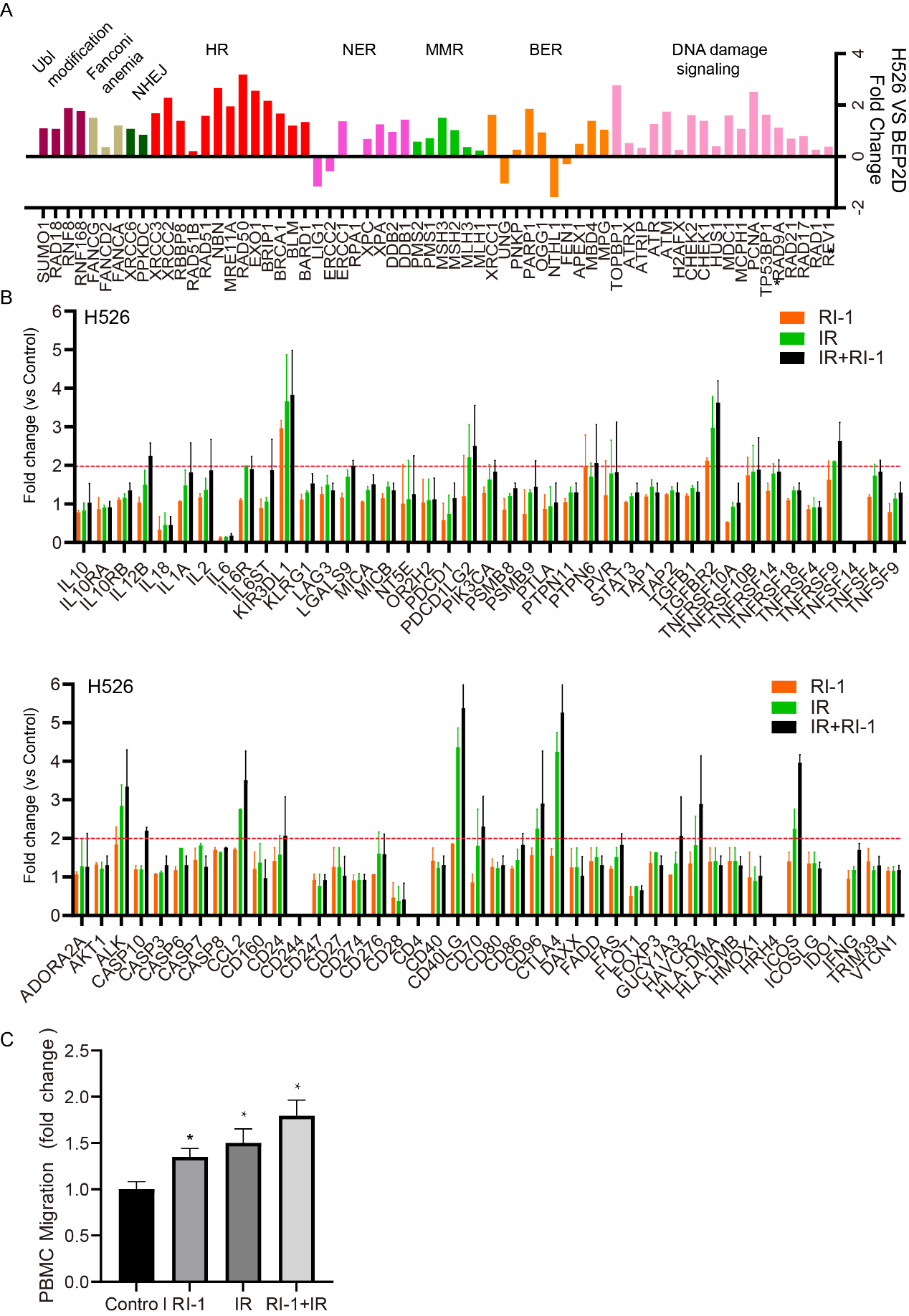


Figure S3. Impact of RAD51 inhibition in H526 cells. **(A)** The relative DDR genes expression profile in H526 cells. **(B)** RAD51 inhibition increased immune checkpoint molecules expressions in H526 cells. H526 cells were pretreated with 10 μM RAD51 inhibitor RI-I for 6h, then followed by 4Gy IR, 12h later cells were harvested and RNAs were extracted for qPCR array. 83 genes related to immune checkpoint were amplified by qPCR. **(C)** RAD51 inhibition promoted the migration PBMCs derived from SCLC patients. Chemotaxis assay was performed using Transwell chambers. PBMC from SCLC patients were seeded into the upper wells, while the lower chambers contained either RPMI medium or conditioned medium from H526 cells treated with RI-1 or in combination with IR for 12h. The results of these experiments are expressed as fold-change vs. control± SEM after 6h incubation (n= 4). Statistical analysis was performed by ANOVA (**P* < 0.05).


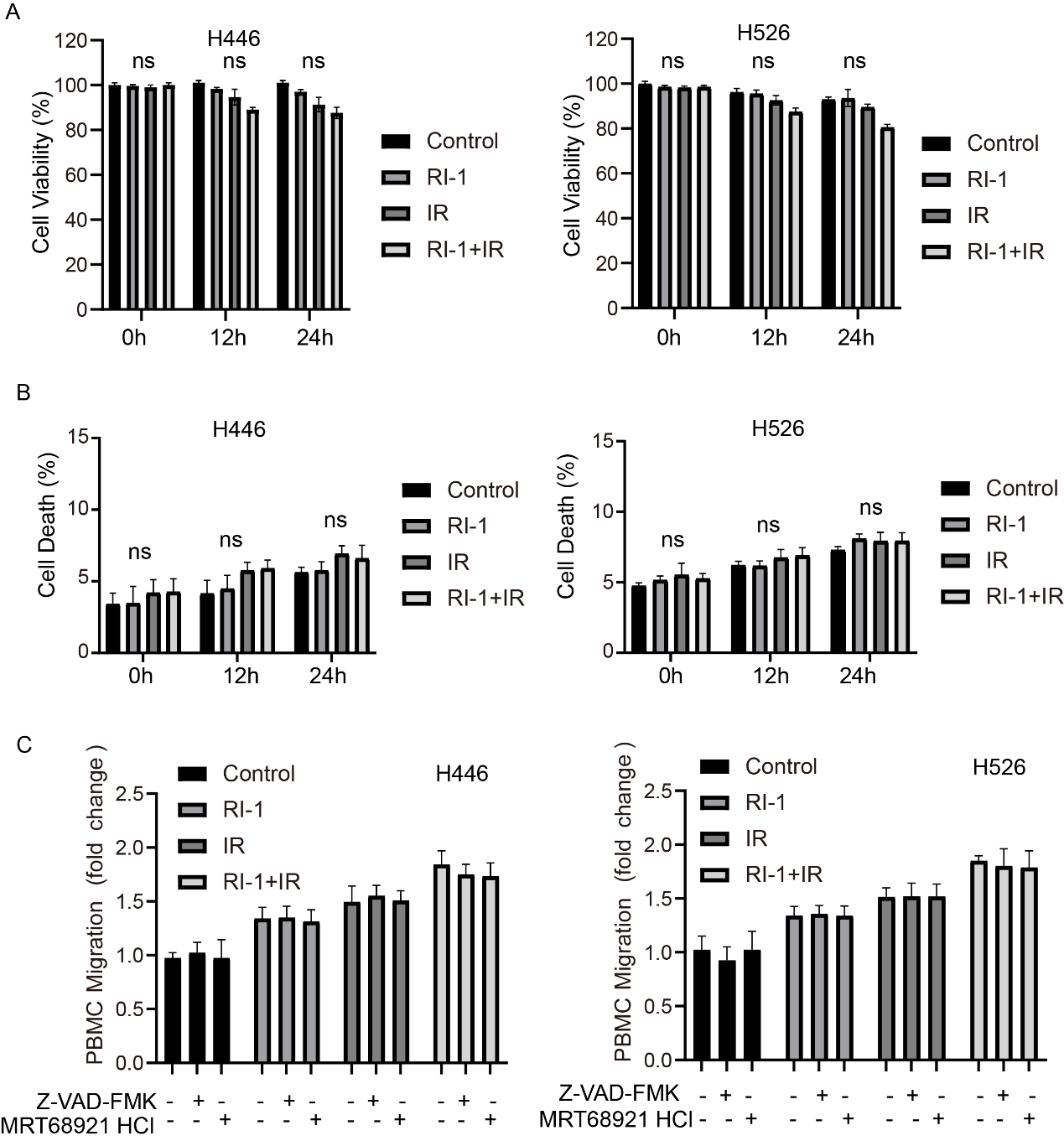


Figure S4. RAD51 mediated PBMC migration effect was not due to cell death. **(A)** H446 or H526 cells were pretreated with 10μM RI-1 and followed by 4Gy IR. The cell viability assay was performed at 0h, 12h and 24h after treatment. The cell viability percentage relative to control was summarized. **(B)** H446 or H526 cells were pretreated with 10μM RI-1 and followed by 4Gy IR. Quantitative analysis of live, early, and late apoptosis and cell death at 0h, 12h and 24h was performed with Muse^@^ Annexin V & Dead Cell Kit. **(C)** H446 or H526 cells were pretreated with 10μM RI-1 and 10μM Z-VAD-FMK or 1μM MRT68921 and followed by 4Gy IR. The PBMC migration was evaluated.


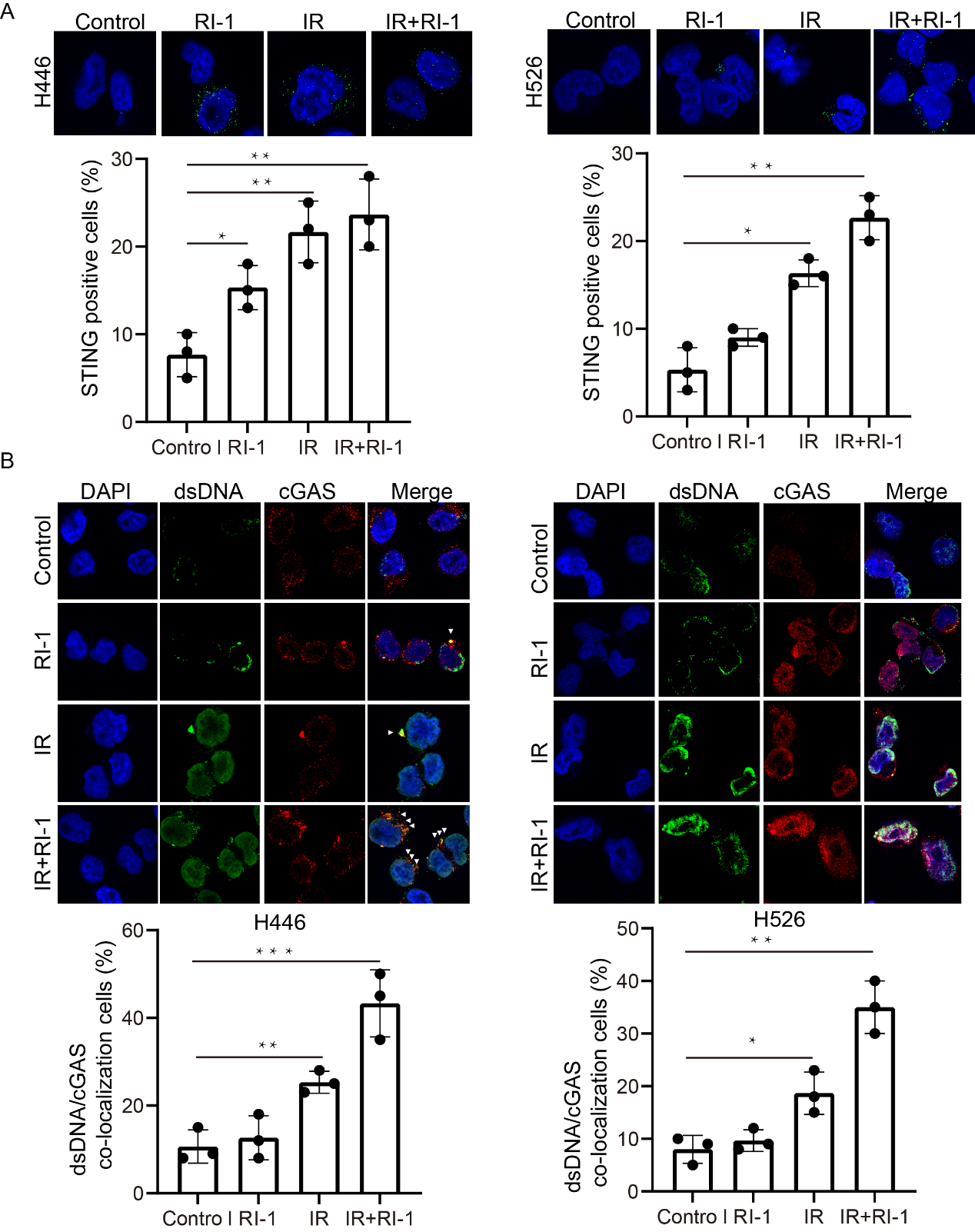


Figure S5. RAD51 inhibition activated the dsDNA-cGAS-STING pathway in SCLC cells. **(A)** H446 or H526 cells were pretreated with 10μM RI-1 and followed by 4Gy IR. Six hours after treatment, the STING expression was stained and summarized. **(B)** H446 or H526 cells were pretreated with 10μM RI-1 and followed by 4Gy IR. Six hours after treatment, the dsDNA and cGAS were co-stained and summarized. * *P* <0.05, ** *P* <0.01, *** *P* <0.001.


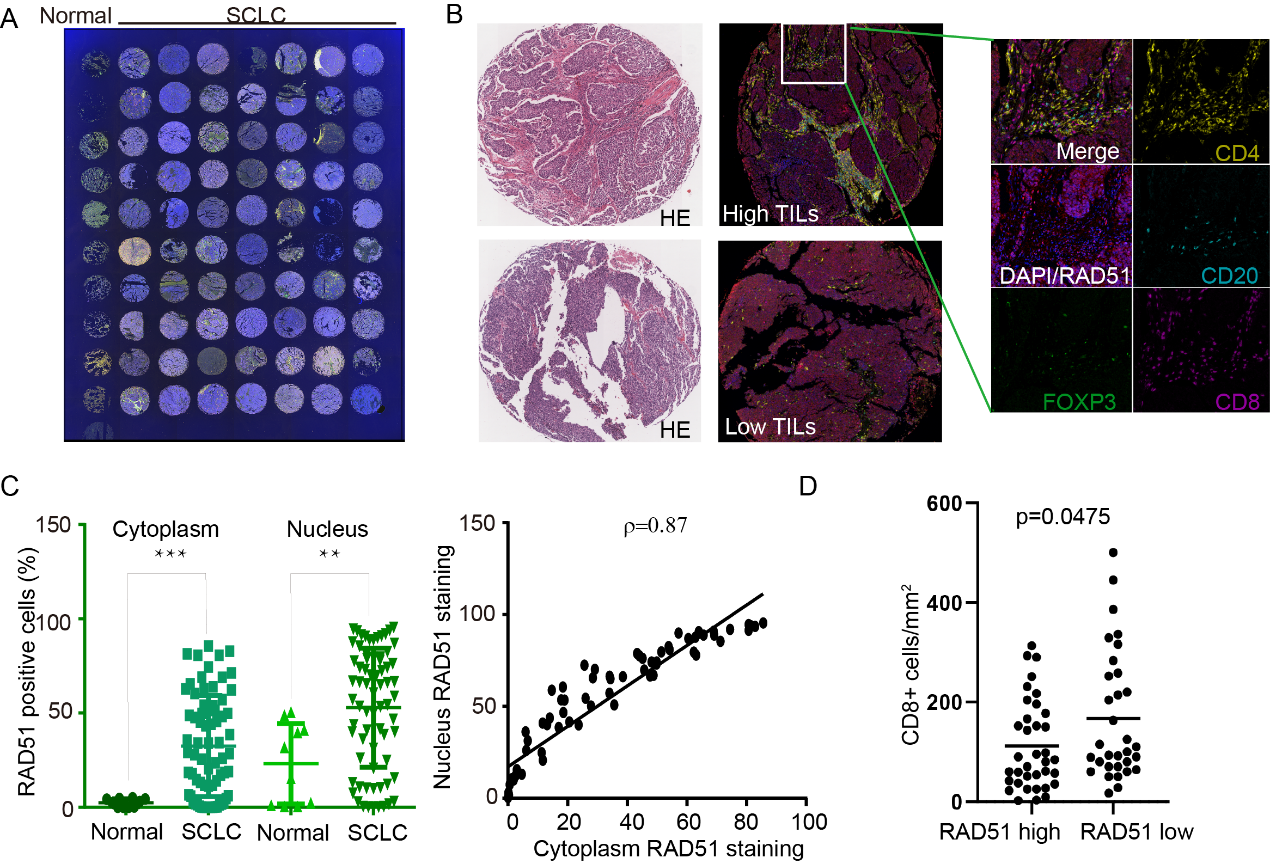


**Figure S6.** Multiplex immunofluorescence staining of RAD51, CD4, CD8, FOXP3 and CD20. (**A)** The overview of the stained SCLC tissue microarray. Normal lung tissues and SCLC tissues were labeled. (**B)** The representative HE staining and immunofluorescence staining images of SCLC tissues with high or low infiltrated lymphocytes. **(C)** Summary of cytoplasmic and nucleus staining of RAD51. The right panel showed the correlation between cytoplasm and nucleus expression of RAD51. (**D)** Summary of CD8+ cell density in SCLC tissues with RAD51 high and low expression.
